# Influence of *N*-Glycosylation on the Morphogenesis and Growth of *Paracoccidioides brasiliensis* and on the Biological Activities of Yeast Proteins

**DOI:** 10.1371/journal.pone.0029216

**Published:** 2011-12-21

**Authors:** Fausto Bruno Dos Reis Almeida, Fernanda Caroline Carvalho, Vânia Sammartino Mariano, Ana Claudia Paiva Alegre, Roberto do Nascimento Silva, Ebert Seixas Hanna, Maria Cristina Roque-Barreira

**Affiliations:** 1 Departamento de Biologia Celular e Molecular e Bioagentes Patogênicos, Faculdade de Medicina de Ribeirão Preto, Universidade de São Paulo, Ribeirão Preto, São Paulo, Brazil; 2 Departamento de Bioquímica e Imunologia, Faculdade de Medicina de Ribeirão Preto, Universidade de São Paulo, Ribeirão Preto, São Paulo, Brazil; 3 Invent Biotecnologia Ltda. ME, Incubadora Supera, Rua dos Técnicos s/n, Universidade de São Paulo, Ribeirão Preto, São Paulo, Brazil; Louisiana State University, United States of America

## Abstract

The fungus *Paracoccidioides brasiliensis* is a human pathogen that causes paracoccidioidomycosis, the most prevalent systemic mycosis in Latin America. The cell wall of *P. brasiliensis* is a network of glycoproteins and polysaccharides, such as chitin, that perform several functions. *N*-linked glycans are involved in glycoprotein folding, intracellular transport, secretion, and protection from proteolytic degradation. Here, we report the effects of tunicamycin (TM)-mediated inhibition of *N*-linked glycosylation on *P. brasiliensis* yeast cells. The underglycosylated yeasts were smaller than their fully glycosylated counterparts and exhibited a drastic reduction of cell budding, reflecting impairment of growth and morphogenesis by TM treatment. The intracellular distribution in TM-treated yeasts of the *P. brasiliensis* glycoprotein paracoccin was investigated using highly specific antibodies. Paracoccin was observed to accumulate at intracellular locations, far from the yeast wall. Paracoccin derived from TM-treated yeasts retained the ability to bind to laminin despite their underglycosylation. As paracoccin has *N*-acetyl-β-d-glucosaminidase (NAGase) activity and induces the production of TNF-α and nitric oxide (NO) by macrophages, we compared these properties between glycosylated and underglycosylated yeast proteins. Paracoccin demonstrated lower NAGase activity when underglycosylated, although no difference was detected between the pH and temperature optimums of the two forms. Murine macrophages stimulated with underglycosylated yeast proteins produced significantly lower levels of TNF-α and NO. Taken together, the impaired growth and morphogenesis of tunicamycin-treated yeasts and the decreased biological activities of underglycosylated fungal components suggest that *N*-glycans play important roles in *P. brasiliensis* yeast biology.

## Introduction

The human pathogen *Paracoccidioides brasiliensis*, a dimorphic fungus, is the causative agent of paracoccidioidomycosis, the most prevalent systemic mycosis in Latin America. The yeast phase is the infectious form and grows at 37°C [1]. The lungs are the primary site of infection, but the yeasts can spread to other organs and cause systemic disease [2].

The yeast cells of *P. brasiliensis* have characteristic features of cell division and budding that allow them to be identified unequivocally by microscopy. One such feature is the co-existence of mother and bud cells during yeast growth, resulting in a wide variety of sizes and shapes and indicating that these cells have an intricate mechanism for regulating polarity during their growth [3], [4], [5], [6]. *P. brasiliensis* does not seem to grow as a single clone with a constant phenotype, and this plasticity may promote the appearance of mutants that are able to evade the host immune response [7].

The cell wall of *P. brasiliensis*, like those of many other fungi, is a network of glycoproteins and polysaccharides that protects the fungal cell from environmental stress [8]. Many of the glycoproteins contain *N*-glycans attached to an asparagine residue within the sequence N-X-S/T, where X denotes any amino acid except proline [9]. *N*-linked glycans are involved in glycoprotein folding, intracellular transport, and protection from proteolytic degradation [10]. The *N*-glycans of *Candida albicans* proteins, for example, have recently been shown to be essential for the integrity of the cell wall as well as for fungus-host interactions [11]. *N*-glycosylation can be altered by a number of natural products, chief of which is tunicamycin (TM). TM belongs to the nucleoside class of antibiotics and inhibits *N*-glycosylation by blocking the transfer of *N*-acetylglucosamine-1-phosphate (GlcNAc-1-P) from UDP-GlcNAc to dolichol-P, thereby decreasing the formation of dolichol-PP-GlcNAc [12]. TM has been used extensively to study the roles of *N*-glycans in glycoprotein maturation, secretion, and function, and its use has been cited in thousands of papers since its discovery in 1973 [13].

Chitin, the second most abundant polysaccharide in nature, is among the best-studied examples of fungal cell wall polysaccharides. It consists of an unbranched homopolymer of 1,4-β-linked *N*-acetyl-d-glucosamine [14] and is present in the cell walls of all fungi studied to date. Its position at specific sites throughout the cell cycle allows it to maintain the overall strength of the wall [15], [16]. In *P. brasiliensis,* chitin is one of the major components of the cell wall and is involved in the morphogenesis and integrity of the wall [17], [18]. The chitinolytic enzyme machinery of fungi consists of chitinases and *N*-acetyl-β-d-glucosaminidase (EC3.2.1.30, NAGase). The functions of chitin-degrading enzymes in fungi include both the use of exogenous chitin as a nutrient source and cell wall remodeling during the fungal life cycle (reviewed by [19]). NAGase cleaves diacetylchitobiose and higher chitin polymers, including chitotriose and chitotetraose, into GlcNAc monomers [14]. The genome of the recently noted Pb18 isolate of *P. brasiliensis* contains at least 4 genes encoding proteins with chitinase domains, as verified by the Broad Institute website (http://www.broadinstitute.org/annotation/genome/paracoccidioides_brasiliensis/FeatureSearch.html). Our laboratory has identified a protein with NAGase activity in a GlcNAc- and chitin-binding fraction of *P. brasiliensis* yeasts, which was named paracoccin [20]. Paracoccin was initially described as a lectin that promotes *P. brasiliensis* adhesion to the extracellular matrix and induces strong and persistent production of TNF-α and nitric oxide (NO) by macrophages [21]; it was only very recently shown to be involved in fungal growth and morphogenesis [22] and to possess NAGase activity [20].

In this study, we have investigated whether *N*-glycans are involved in the cell growth and morphogenesis of *P. brasiliensis* and the impact of underglycosylation on the activities of fungal proteins. We found that *N-*glycans are important for the growth and morphogenesis of yeasts and for the NAGase and macrophage-activating functions of yeast proteins.

## Results

### Tunicamycin inhibits the growth of *P. brasiliensis* without affecting yeast viability

A preliminary *in vitro* dose-response assay demonstrated that *P. brasiliensis* growth is dramatically inhibited by addition of tunicamycin (TM) at concentrations greater than or equal to 15 µg*/*mL (data not shown) to the yeast cultures. TM was used at a final concentration of 15 µg/mL in all subsequent assays.

**Figure 1:**
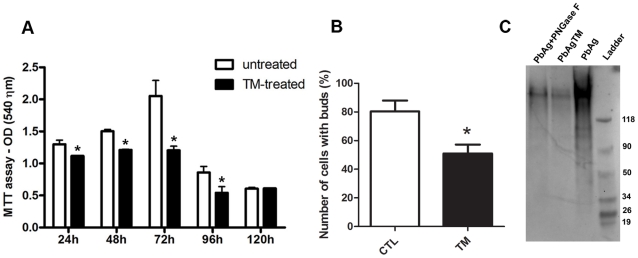
Effect of tunicamycin (TM) on the growth and budding of *P. brasiliensis* yeast cells. MTT assay of yeast cells cultivated in YPD medium in the absence (white bars) or presence (black bars) of 15 µg/mL TM (A). Number of cells with buds in YPD medium in the absence (white bar, CTL) or presence (black bar, TM) of 15 µg/mL TM for 72 h at 37°C, and analyzed by optical microscopy and counted using neubauer chamber (B). Western blotting with ECL glycoprotein detection of the crude extract fully glycosylated (PbAg), crude extract underglycosylated (PbAgTM) and crude extract fully glycosylated treated with N-glycanase PNGase F (PbAg + PNGase F) (C). Data are representative of 3 independent assays. * p<0.05 for comparison between CTL and TM.

The time course of growth in culture, as determined by the MTT assay, was compared between TM- and vehicle-treated yeasts. Figure 1A shows that the addition of TM to the culture inhibited fungal growth by 45% after 72 h compared with the vehicle-treated culture (untreated). No significant inhibition was detected at other timepoints. Similar patterns of growth inhibition were observed when yeast growth was measured by CFU counting or OD readings (data not shown). To determine whether TM treatment of *P. brasiliensis* induces death, viability assays were performed using fluorescein diacetate/ethidium bromide staining. Treated and untreated cultures contained similar proportions of viable yeasts regardless of TM treatment, and all cultures were >85% viable.

We assessed the average number of cells with buds in the yeast cultured in the presence or absence of TM. We counted the budded or unbudded yeast cells using optical microscopy with a Neubauer chamber. The results are shown in Figure 1 B. The average number of budding cells was 80 per 100 untreated cells and 51 per 100 TM-treated cells (p = 0.0067; t-test).

In order to verify whether the TM treatment results in the inhibition of glycosylation in *P. brasiliensis* yeast cells, we carried out western blot analysis of the fully glycosylated crude yeast extract (100 µg of PbAg), the under-glycosylated crude extract (PbAgTM), and the fully glycosylated crude extract after PNGase F digestion. The enhanced chemiluminescence (ECL) kit for detecting glycoprotein strongly revealed several components in the PbAg, whereas only 1 high-molecular-weight glycoprotein was detected at low concentrations in the extracts obtained from TM-treated yeasts or those digested with PNGase (Figure 1 C).

### Morphological changes in tunicamycin-treated *P. brasiliensis* yeasts

**Figure 2:**
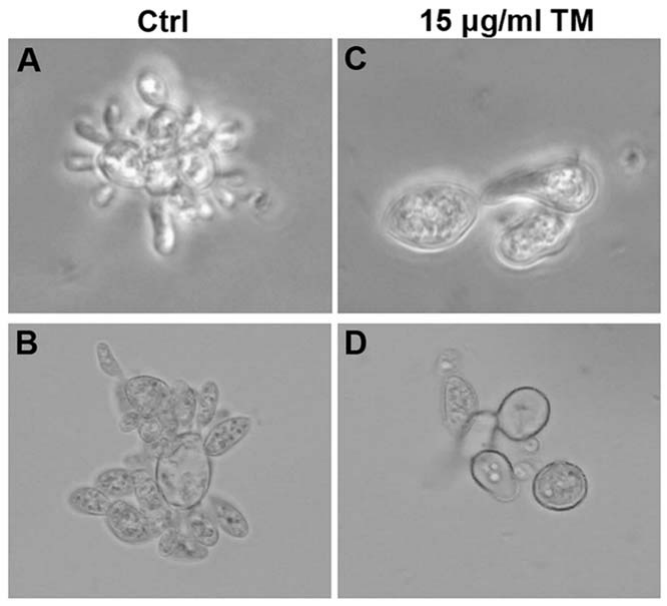
Effect of tunicamycin (TM) on the morphology of *P. brasiliensis* yeast cells. *P. brasiliensis* was grown in YPD medium in the absence (Ctrl), or in the presence of 15 µg/mL TM for 72 h at 37°C and analyzed by optical microscopy (A and C) and phase-contrast microscopy (B and D).

**Figure 3:**
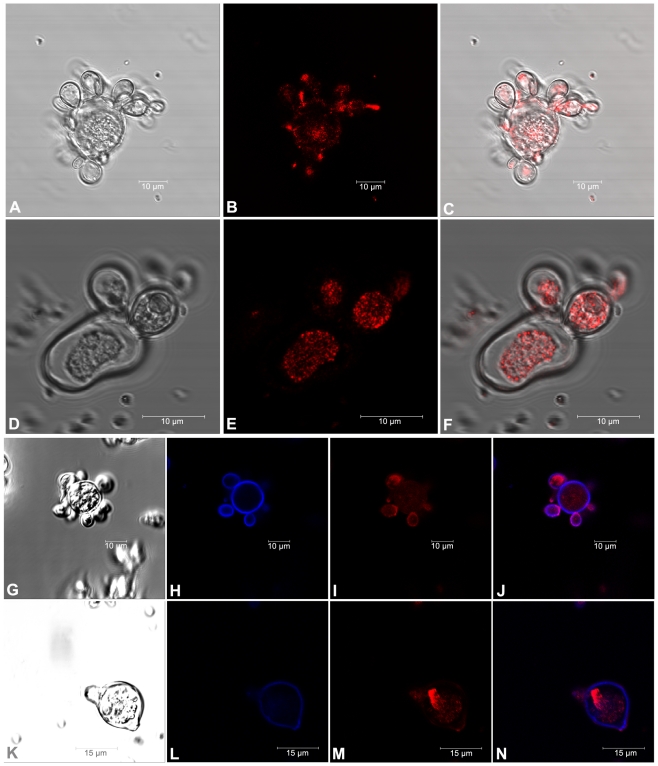
Effect of tunicamycin (TM) on the distribution of paracoccin in *P. brasiliensis* yeasts. Fluorescence labeling with anti-paracoccin IgY was evenly distributed over the yeast surface. In some budding regions, a more intense labeling was detected for untreated *P. brasiliensis* yeast cells (B, C, I and J) compared with those treated with TM (E, F, M and N), which were more centrally labeled. Panels H and L show fluorescence labeling chitin, while panels B, E, I and M show fluorescence labeling paracoccin. C is the merge of A and B; F is the merge of D and E; J is the merge of G, H and I; and N is the merge of K, L and M.

To examine the effects of inhibition of *N*-glycosylation on fungal morphology, *P. brasiliensis* yeasts were treated with 15 µg/mL TM, cultivated for 72 h, and examined by optical and phase-contrast microscopy (Fig. 2). The yeast cells showed typical microscopic features (Fig. 2A and 2B) when grown in the absence of TM. By contrast, the TM-treated yeasts were smaller than untreated cells and exhibited a drastic reduction in cell budding (Fig. 2C and 2D). Similar results are shown in Figure 3. The reduced cell budding in TM-treated yeasts is consistent with the growth inhibition of TM-treated yeasts illustrated in Figure 1.

### Impaired intracellular distribution of paracoccin in tunicamycin-treated yeasts

Because *N*-glycosylation is involved in cellular protein secretion, we investigated the effect of TM treatment on the cellular distribution of paracoccin, a glycoprotein detected in the *P. brasiliensis* yeast cultures. Yeast cells were stained for paracoccin using anti-paracoccin IgY and analyzed by confocal microscopy (Fig. 3). Paracoccin was detected in both TM-treated and untreated yeasts, but the treatment altered the cellular protein distribution and fluorescence pattern. In control yeasts, paracoccin was detected throughout the entire cell, showing regions of mild punctate pattern of fluorescence in the cytoplasm. Nonetheless, fluorescence was stronger and homogeneous in the growing extremities of the buds (Fig. 3B and 3C). In TM-treated yeasts, paracoccin staining displayed a punctate, reticular pattern suggesting an accumulation of the protein within the endoplasmic reticulum (ER) and cytoplasmic vesicles (Fig. 3E and 3F). The shift in paracoccin localization in response to TM treatment is clearer when yeast cells are co-stained with calcofluor to highlight the cell-walls. In control cells, paracoccin is mostly localized to regions adjacent to cell walls (Fig. 3I and 3J), suggesting targeting to the cell surface. Consistently, cell surface staining of paracoccin is greatly lost upon TM-treatment (Fig. 3M and 3N).

### Underglycosylation of yeast proteins does not affect their binding to laminin

**Figure 4:**
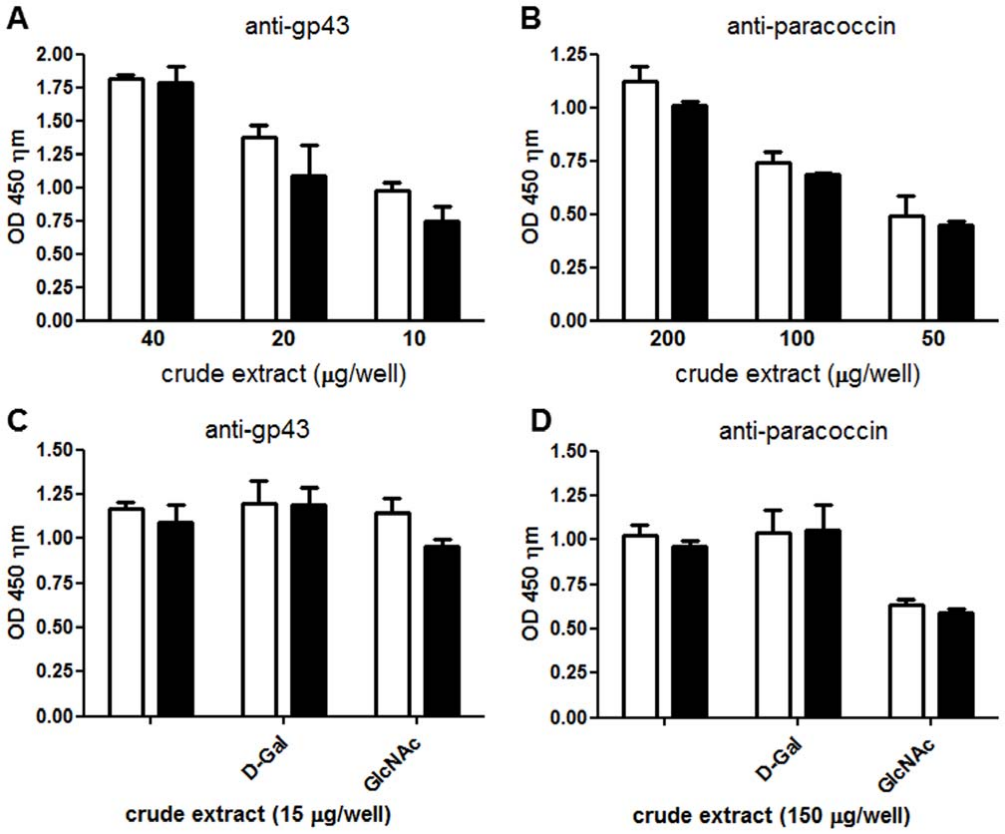
Effect of tunicamycin (TM) on binding of paracoccin and gp43 to laminin. Binding of paracoccin and gp43 to laminin was assayed by incubating different amounts of fully glycosylated and underglycosylated crude extracts (white bars and black bars, respectively) of *P. brasiliensis* in laminin-coated (0.5 µg/well) microplate wells, followed by incubation with biotinylated monoclonal antibody anti-gp43 (A) or with biotinylated IgY anti-paracoccin (B). The reactions were developed with neutravidin–peroxidase conjugate and H_2_O_2_/OPD substrate. Optical density (OD) readings at 450 nm show dose-dependent binding (A and B). Inhibition assays for gp43 (C) and paracoccin (D) binding to laminin were performed in the presence of 10 mM d-galactose (d-Gal) or N-acetyl-glucosamine (GlcNAc). Data are representative of 3 independent assays.

We next investigated whether underglycosylation of paracoccin affected its binding to laminin. In parallel, the binding of an additional yeast glycoprotein, gp43, was also examined. Different amounts of fully glycosylated and underglycosylated crude extracts (*Paracoccidioides brasiliensis* soluble antigen; PbAg) from yeasts that had been cultured for 72 h in the absence or presence of TM, respectively, and then disrupted, were incubated, at concentrations of 0.1–2 mg/mL, in microplate wells coated with 0.5 µg/well laminin. The laminin-bound gp43 and paracoccin were detected by their respective antibodies. Dose-response curves for each protein were plotted by binding PbAg in concentrations of 0.1–0.4 mg/mL for gp43 detection (Fig. 4A) and 0.5–2.0 mg/mL for paracoccin detection (Fig. 4B). These different ranges were consistent with the different levels of these components in *P. brasiliensis* yeast cells, since gp43 is much more abundant than paracoccin. The levels of binding of fully glycosylated and underglycosylated proteins were similar between paracoccin and gp43, indicating that their interactions with laminin were not affected by the TM-mediated loss of *N*-glycans. The interaction of paracoccin with laminin was, however, selectively inhibited by GlcNAc (Fig. 4D), reflecting the dependence of this interaction on its carbohydrate recognition properties and contrasting with the gp43-laminin interaction, which was independent of added sugars (Fig. 4C).

### Tunicamycin-induced underglycosylation reduces the NAGase activity of lysates from *P. brasiliensis* yeasts

**Figure 5:**
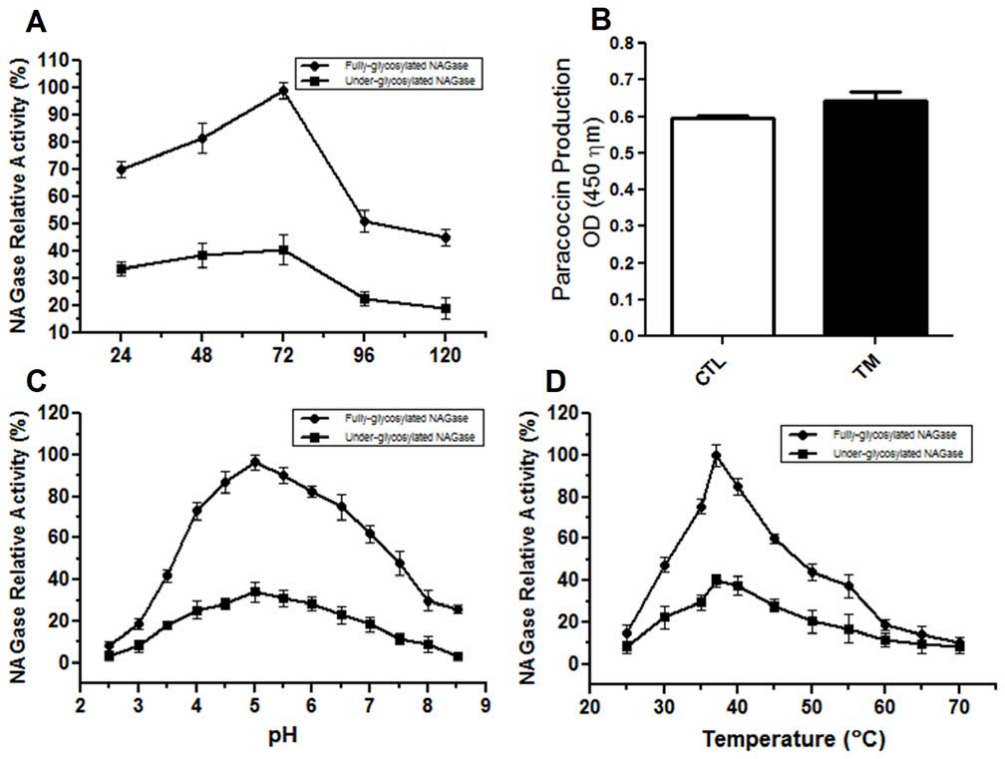
Effect of tunicamycin (TM) on the NAGase (N-acetyl-β-d-glucosaminidase) activity of *P. brasiliensis* yeasts. *P. brasiliensis* yeasts cultured in YPD medium in the absence or presence of 15 µg/mL TM were disrupted to obtain crude extracts. The time-course profile of NAGase activity, as detected by the degradation of *p*NP-GlcNAc substrate (A); the effect of TM on the expression of paracoccin, a *P. brasiliensis* protein with NAGase activity, shown by ELISA using anti-paracoccin IgY and measured at 450 nm (B); and profiles of optimal pH (C) and temperature (D) for the fully glycosylated and underglycosylated NAGases. Maximum activity was standardized as 100% and NAGase activities were measured at 405 nm. Data are representative of 3 independent assays.

Equal amounts of total protein derived from disrupted yeasts cultured 24–120 h after addition of TM or vehicle were assayed for NAGase activity. Samples from TM-treated yeasts displayed NAGase activity at least 2-fold lower than those from control yeasts did. Both the maximum NAGase activity and the greatest difference (3-fold) between samples derived from TM-treated and control yeasts were observed at the 72 h timepoint (Fig. 5A). As the decreased NAGase activity detected in the material derived from TM-treated yeasts could have been due to decreased synthesis of paracoccin rather than to an effect of decreased *N*-glycosylation on NAGase activity, we used the highly specific anti-paracoccin IgY to compare the levels of paracoccin expression in the samples obtained from TM-treated and control yeasts. We found that the levels were very similar between TM-treated and control samples (Fig. 5B), suggesting that paracoccin, which has NAGase activity, is equally well expressed in the presence or absence of TM. Although paracoccin is not responsible for all *P. brasiliensis* NAGase activity, our results indicate that protein underglycosylation accounts for the reduction of total NAGase activity in TM-treated *P. brasiliensis* yeasts.

To evaluate the influence of protein underglycosylation on various aspects of fungal enzymatic activity, preparations of total soluble *P. brasiliensis* components (PbAg) obtained by disruption of TM-treated or untreated yeasts and containing similar protein concentrations were assayed for NAGase activity. As shown in Figure 5, both fully glycosylated and underglycosylated PbAg preparations showed maximal NAGase activity under similar conditions, i.e., at pH 5 (panel C) and 37°C (panel D). Under these optimal conditions, fully glycosylated PbAg demonstrated 3–5 times more NAGase activity than did underglycosylated PbAg.

In addition, the kinetic parameters of the NAGase activity were determined for fully glycosylated and underglycosylated preparations. The Michaelis–Menten constant (K_m_) was determined from non-linear-regression analysis of data obtained by measuring the rate of ρ-nitrophenyl-β-*N*-acetylglucosaminide (ρNP-GlcNAc) hydrolysis (0.01–0.1 mM). Table 1 shows that the K_m_ of the NAGase was substantially lower for the fully glycosylated PbAg (0.22 mM) than for the underglycosylated PbAg (0.36 mM). In addition, the V_max_ of the fully glycosylated NAGase (1.18 U/mg) was higher than that of the underglycosylated NAGase (2.4 U/mg). Taken together, these results suggest that *N*-glycosylation is important for stabilizing the structure of the NAGase catalytic site. However, further studies are necessary to determine whether *N*-glycosylation influences the binding of the substrate to the catalytic site or merely the access of the substrate to the catalytic site via microdiffusion.

**Table 1:** Partial kinetic characterization of N-acetyl-β-d-glucosaminidase activity (NAGase) produced by *P. brasiliensis* in the absence or presence of tunicamycin.

NAGase	K_m_ (mM)	V_max_
Fully glycosylated	0.22±0.01	2.40±0.15
Underglycosylated	0.36±0.04	1.18±0.09

Data are representative of 3 independent assays.

### Underglycosylation of yeast proteins inhibits their induction of TNF-α production by macrophages

**Figure 6:**
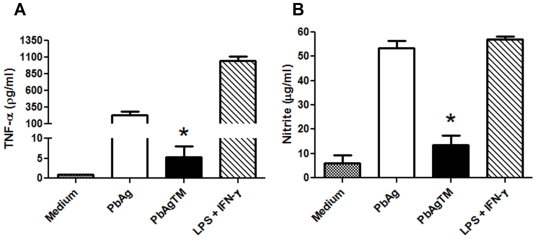
Effect of underglycosylation on the yeast proteins ability to induce inflammatory mediator production in macrophages. Adherent cells from the peritoneal cavities of C57BL/6 mice were stimulated *in vitro* for 48 h with crude extracts of *P. brasiliensis* yeasts with (PbAgTM) or without (PbAg) TM treatment. Stimulation with LPS+IFN-γ was used as a positive control. The culture supernatants were assayed for TNF-α (A) and NO (B) levels. Data are representative of 3 independent assays. * p<0.05 between PbAgTM and PbAg.

We also compared the abilities of fully glycosylated and underglycosylated PbAg to induce murine macrophages to produce TNF-α and NO (Fig. 6A and 6B, respectively). High levels of TNF-α and NO were released by peritoneal macrophages stimulated with fully glycosylated PbAg, as previously reported for paracoccin [20], [21]. In contrast, significantly less TNF-α and NO were produced by macrophages stimulated with underglycosylated PbAg. Tunicamycin was added directly to the murine peritoneal macrophage suspension as a negative control and had no effect on cytokine production (data not shown).

## Discussion

This study shows that *N*-glycans are required for the normal growth and morphogenesis of *P. brasiliensis* yeasts, as demonstrated by tunicamycin (TM) treatment of yeast cells. In addition to the effects on the whole yeast, the TM-induced underglycosylation inhibited most of the known biological activities of the fungal protein paracoccin, contained in the crude cell extract. Paracoccin, which is normally secreted by yeasts, was found to accumulate at intracellular sites in TM-treated yeasts.

Tunicamycin has been used extensively for studies of the roles of *N*-glycans in protein secretion and function. TM treatment prevents the first step in the *N*-glycosylation of proteins, i.e., the transfer of the lipid-linked oligosaccharide precursor to an asparagine residue immediately after the nascent protein chain has entered the lumen of the rough endoplasmic reticulum (RER). Because all further steps of *N*-glycosylation are based on the transfer of a pre-made oligosaccharide to the protein backbone, in principle TM blocks all *N*-glycosylation (reviewed by [23]). Its profound effect on this post-translational protein modification makes the impact of TM on a particular experimental design somewhat unpredictable. TM treatment may also inhibit protein biosynthesis, although to a lesser degree than the inhibition of glycosylation for the majority of systems, and it is necessary to consider this effect when designing and interpreting experiments using TM treatment. In our study, the intracellular accumulation of the yeast glycoprotein paracoccin in TM-treated organisms strongly suggests that the protein synthesis was not significantly affected. On the other hand, the accumulation of these proteins in what appears to be the endoplasmic reticulum (ER) compartment could indicate a profound alteration of protein secretion that may be attributed to the absence of *N*-glycosylation.

As more than half of all proteins in nature, including those from fungi, are estimated to be glycosylated, and three-quarters of all glycoproteins are thought to contain *N*-linked glycans [24], it is not surprising that some cellular processes are altered in *P. brasiliensis* yeasts exposed to TM. The TM-induced disruption of subcellular protein localization that we observed was also demonstrated to occur in a study on the importance of glycosylation for the subcellular localization of 2 mitochondrial glycoproteins of *Saccharomyces cerevisiae* [25]. In addition, we have demonstrated that yeast growth and morphogenesis were also affected relative to the control by TM at concentrations greater than 15 µg/mL; this observation is consistent with previous assessments of the effect of TM on other fungi including *Trichoderma harzianum* [26], *Cryptococcus albidus* [27], *Aspergillus oryzae* [28], and *Cryptococcus flavus* [29]. Indeed, the range of organisms and glycoproteins investigated for susceptibility to TM treatment is so diverse that it is not surprising that the reported consequences of inhibition of *N*-glycosylation are also wide-ranging [23].

We also examined the effect of TM treatment on some known biological activities of paracoccin that was abnormally concentrated at intracellular sites in TM-treated yeasts. The underglycosylated and fully glycosylated cell extracts were assessed for NAGase and laminin-binding activities. Paracoccin has both of these activities [20], whereas gp43 only binds to laminin [30], [31]. The effect of deglycosylation on enzyme structure is most often observed by changes in thermal or pH stability and kinetic parameters [29]. While we observed no difference in the optimal pH and temperature conditions between underglycosylated and fully glycosylated paracoccin, the decreased NAGase activity of underglycosylated PbAg suggests that *N*-glycans play a significant role in the enzymatic activity. Therefore, the lower NAGase activity may be involved in the impaired growth and morphogenesis of TM-treated *P. brasiliensis* yeasts. This hypothesis is consistent with the fact that chitin, a GlcNAc polymer that is the major constituent of the yeast wall, is the natural substrate for NAGase.

Another point to be considered is that NAGase works in synergism with chitinases. Thus, since NAGase depends on the end products from the action of chitinase on the chitin polymer, the influence of glycosylation inhibition could be indirect. However, cell wall dynamics are not a simple process related only to chitin polymers. There are many wall-associated enzymes such glucanases, peptidases, and glycosyltransfarases, which are glycoproteins, and thus, are subject to co-regulation and the influence of glycosylation [32].

Deficiencies in chitin degradation may be responsible for the impaired yeast morphogenesis and reduced budding that were observed in TM-treated yeasts, which would be consistent with the previous demonstration that paracoccin is predominantly detected in the budding regions of the yeast and plays an important role in its growth [22]. However, we did not observe significant differences in chitin levels followed CFW staining (Figure 3) in TM-treated and non-treated cells. The metabolism of chitin in the fungal cell wall is a dynamic process [33] and further studies to analyze the kinetics of chitin formation and degradation in *P. brasiliensis* yeast cells will be necessary to correlate chitin content and yeast morphogenesis.

The binding of paracoccin and gp43 to laminin was similar for underglycosylated and fully glycosylated cell extracts, showing that glycosylation of either protein does not influence the binding to laminin. The fact that binding of paracoccin but not gp43 to laminin was inhibited by GlcNAc demonstrates the different mechanisms by which each protein interacts with laminin; as previously reported, the interaction of gp43 with laminin is not mediated by carbohydrate recognition [21]. Moreover, the fact that the NAGase activity but not the lectin activity of paracoccin is affected by the TM-induced underglycosylation reinforces our previous hypothesis that different domains of paracoccin are responsible for each property [20].

*N*-glycosylation of *P. brasiliensis* yeasts may also interfere with the fungal relationship with mammalian host cells, as indicated by the decreased TNF-α and NO production by murine macrophages stimulated *in vitro* with underglycosylated PbAg rather than fully-glycosylated PbAg. Although induction of TNF-α and NO is another property of paracoccin [21], exerted through its lectin domain, we cannot yet exclude the involvement of other fungal components in the macrophage response. Conversely, the underglycosylation may implicate in the absence of some sites for recognition of fungal carbohydrates by macrophage lectins, a fact that would impair the signaling that leads to production of TNF-α and NO (reviewed by [34]).

The results of this study demonstrate that *N*-glycosylation of *P. brasiliensis* proteins is crucial for many fungal biological processes as well as for the normal interaction of the yeast with host cells. As underglycosylation of fungal proteins interferes with activities that are relevant to the pathogenesis of paracoccidioidomycosis, we hypothesize that therapies directed at *N*-glycans may be an alternative to combat the fungal infection.

## Materials and Methods

### Ethics Statement

This study was approved by the Ethics Committees and the Institutional Review Board of the University of Sao Paulo [ process number 145/2005].

### Strain and culture conditions

*P. brasiliensis* Pb18, a highly virulent isolate, was maintained in the yeast form in Fava Netto semi-solid medium and incubated at 36°C, as previously described [35]. To ensure the maintenance of Pb18 virulence, serial passages in BALB/c mice were performed before the isolate was used in experiments. For yeast growth, cultures were incubated in liquid YPD medium (2% peptone, 1% yeast extract, and 2% glucose) at 36°C on a rotary shaker at 100 rpm for 72 h. Yeast cells were incubated in liquid YPD medium with different concentrations of TM for 5 days at 36°C, and the culture growth was monitored daily by counting the cells to determine the optimal TM concentration and duration of treatment for further analysis. The TM concentration used in our assays was 15 µg/mL (prepared in 20 mM of NaOH), which was sufficient to inhibit cell growth relative to TM-free control cells.

The crude extracts were obtained by sonication, as previously described [20]. To verify if the TM treatment results in inhibition of glycosylation in *P. brasiliensis* yeast cells, samples of the crude extract obtained from yeasts cultured in the presence or absence of TM were analyzed by western blot developed by ECL glycoprotein detection, as described in the next section of this chapter.

To verify the effect of TM in the yeast budding, we assessed the average number of cells with buds found in yeasts that were cultured in the absence or presence of TM, counting was carried out in a neubauer chamber by optical microscopy, considering as budding cells, the yeasts that showed at least one bud.

### SDS-PAGE/western blot and ECL glycoprotein detection

Sodium dodecyl sulfate–polyacrylamide gel electrophoresis (4%–20%; Genscript, USA) was used according to the manufacturer's protocol, using Tris-Mops-SDS running buffer in a Mini-Protean 3 electrophoresis cell (Bio-Rad Laboratories Richmond, CA, USA). Pre-stained standards (PageRuler; Fermentas, Ontario, Canada) were used for estimating the molecular weights of the sample proteins. Polyacrylamide gel was electrotransferred to a nitrocellulose membrane (Hybond-C Extra; Amersham Biosciences), which was developed by the Amersham ECL glycoprotein detection system, according to the manufacturer's instructions. The membrane was incubated in PBS for 10 min, and then incubated in the dark in 10 mM sodium metaperiodate for oxidizing the carbohydrate groups present. The membrane was then washed with PBS and incubated with biotin hydrazide for 60 min, in order to label the protein with biotin on carbohydrate residues, and washed again with PBS. Non-specific interactions were blocked overnight at 2°C, and then washed with PBS. The membrane was incubated with streptavidin-horseradish peroxidase conjugate prepared in PBS for 30 min, and then washed with PBS. Finally, glycoprotein detection was performed with ECL (Enhanced Chemo-Luminescence).

### Cell viability and proliferation assays

The viability of fungal suspensions was determined by fluorescein diacetate/ethidium bromide staining. Only the cultures that were greater than 85% viable were used. The effect of TM on cell proliferation was determined by colony counting, optical density (OD), and MTT assays. *P. brasiliensis* yeast cells were suspended in YPD medium at a density of 1×10^6^ cells/mL, and TM was added to a final concentration of 15 µg/mL. TM-free control cells were supplemented with 20 mM NaOH (vehicle). After incubation at 36°C in an orbital shaker (100 rpm) for 2–5 days, the cell growth was analyzed using the tetrazolium salt 3-[4,5-dimethylthiazol-2-yl]-2,5-diphenyltetrazolium bromide (MTT, Sigma) with a colorimetric assay for measuring mitochondrial activity. *P. brasiliensis* yeast cells were cultivated with TM for 72 h at 36°C (100 rpm) in 12-well tissue culture plates with the addition of 10 µL/well of MTT (5 mg/mL). After incubation for 4 h at 36°C, the medium containing MTT was partially removed, dimethyl sulfoxide (100 µL/well) was added to solubilize the formazan product, and the OD was measured at 540 nm.

### Chicken immunization and processing of anti-paracoccin antibodies

Antibodies were produced in chickens as previously described [36] with slight modifications as follows. Immunization was carried out by intramuscular injection of 80 µg of chitin-purified NAGase (paracoccin) emulsified in Freund's complete adjuvant (Sigma Chemical Co.). Serum was collected for the analysis of specific antibodies production by immunoblotting. Booster immunizations with the antigen emulsified in Freund's incomplete adjuvant (Sigma Chemical Co.) were performed 2, 4, and 6 weeks post-inoculation. The eggs were harvested and kept at 4°C until purification of specific IgY. The soluble fraction of the egg yolk was prepared as previously described [36]. The eggs were washed with water and broken, and the content was poured onto a Whatman #1 filter paper in order to separate the yolk from the white. In the next step, a pool of yolks was filtered to retain the chorioallantoic membrane. Yolk contents were diluted 1∶10 in ultrapure water. After 6 h of incubation at 4°C with gentle agitation, high-density lipids were pelleted by centrifugation (10,000× *g*, 25 min, 4°C) and the IgY-containing supernatant was collected. IgY purification was performed by precipitation with 40% ammonium sulfate. Briefly, the soluble fraction was collected and incubated at 4°C under constant agitation, and the ammonium sulfate solution was added drop by drop. The suspension was then centrifuged at 10,000× *g* for 10 min, and the supernatant was discarded. The pellet was resuspended in PBS and precipitated twice more using the same procedure. The final pellet was resuspended in PBS and dialyzed against PBS using YM-10 membrane (Amicon Div W. R. Grace Co. Danvers, MA, USA) to remove any trace of ammonium sulfate.

The anti-paracoccin biotinylation was performed as previously described by Coltri et al., 2006, with slight modifications. One milliliter of 20 mM sodium meta-periodate in sodium acetate buffer (0.1 M, pH 5.5) was incubated with 2 mg of anti-paracoccin for 1 h at 4°C in the dark. The oxidation reaction was stopped by adding glycerol to a final concentration of 15 mM for 5 min at 0°C. After dialysis against sodium acetate buffer (0.1 M, pH 5.5), the solution of anti-paracoccin with oxidized carbohydrate moieties was incubated with biotin hydrazide to a final concentration of 5 mM (Pierce Chemical Co.). The resulting mixture was allowed to react under stirring for 3 hours at room temperature. Biotinylated anti-paracoccin was dialyzed against water in a Centricon system YM-50 (Millipore Centricon®), and stored at −20°C.

The specificity of the anti-paracoccin antibody has previously been demonstrated by Ganiko et al, 2007. Briefly, anti-paracoccin antibodies were adsorbed in paracoccin (500 ng/dot) immobilized on nitrocellulose membrane. The reactivity of the non-absorbed antibodies and the absorbed and eluted antibodies with paracoccin was evaluated by confocal fluorescence microscopy. Only the absorbed antibodies gave a positive fluorescence reaction.

### Enzymatic assay and protein determination

NAGase activity was assayed by mixing 100 µL of 5 mM *p*NP-GlcNAc (Sigma), 350 µL of 0.1 M sodium acetate buffer (pH 6.0), and 50 µL of the tested sample, and the absorbance at 405 nm was measured as previously described [37], [38], [39]. The tested samples (fully glycosylated and underglycosylated) were corrected to the same final protein concentration. One enzymatic unit was defined as the amount of enzyme required to produce 1 µmol of ρ-nitrophenol per minute under optimal reaction conditions. Protein concentrations were determined by the BCA method (Sigma) using bovine serum albumin as the standard.

Measurement of the amounts of both NAGases (fully glycosylated and underglycosylated) was verified by an antibody-binding assay using the anti-paracoccin IgY antibody (anti-NAGase) biotinylated using the EZ-Link Sulfo-NHS Biotin Kit (Pierce Chemical Co.). Each well of a 96-well microplate (MaxiSorp FluoroNunc, Roskilde, Denmark) was coated with 100 µg of the *P. brasiliensis* crude extract (PbAg) diluted in 50 mM carbonate-bicarbonate buffer (50 µL/well) and incubated for 18 h at 4°C. The plate was incubated first with the biotinylated anti-paracoccin antibody and then with streptavidin-HRP (horseradish peroxidase) (BD Biosciences Pharmingen). The reaction was developed with TMB substrate and the absorbance at 450 nm was read using a Multiskan microplate reader (MMC/340P, version 2.20, Labsystems, Helsinki, Finland).

### N-acetyl-β-d-glucosaminidase characterization

The effect of pH on the enzymatic activity was determined by varying the pH of the reaction mixtures using 0.1 M phosphate citrate buffer (pH 2.5–5.0), 0.1 M sodium acetate buffer (pH 5.5–7.5), or 0.1 M sodium phosphate buffer (pH 8.0–8.5). The effect of temperature on the enzymatic activity at optimum pH was determined by varying the temperature of the reaction in the range 25–70°C. The Michaelis-Menten constant (K_m_) was determined from the Lineweaver-Burk representation of the data obtained by measuring the initial rate of *p*NP-GlcNAc hydrolysis using a range of 0.1–1.0 mM.

### Confocal microscopy

For colocalization studies, paraformaldehyde-fixed yeast cells (3×10^6^) were incubated with 100 µg/mL of biotinylated anti-paracoccin antibody in gelatin-PBS-T (phosphate buffered saline with 1% gelatin and 0.5% Tween) for 1 h at room temperature. Samples were then incubated for 1 h in the dark with a mixture of 10 µg/mL calcofluor to visualize chitin and 10 µg/mL Alexa Fluor 594-conjugated streptavidin to visualize anti-paracoccin antibody. Preparations were analyzed using a laser scanning confocal microscope Leica TCS SP5 (Carl Zeiss, Jena, Germany) with 40× NA 1.3 and 63× NA 1.4 plan apochromatic objectives.

For phase-contrast microscopy studies, the samples were observed with an immunofluorescence microscope Leica DMI 4000B.

### Binding of fully glycosylated and underglycosylated glycoproteins (paracoccin and gp43) to laminin

Each well of a 96-well microplate (MaxiSorp FluoroNunc, Roskilde, Denmark) was coated with 0.5 µg of laminin (derived from mouse Engelbreth-Holm-Swarm sarcoma) in carbonate buffer (pH 9.6) overnight at 4°C. After washing and incubation with a blocking solution (3% gelatin), different doses of the crude extract from *P. brasiliensis* were added to the wells. Following incubation at room temperature for 90 min, the wells were washed and incubated with biotinylated anti-paracoccin or anti-gp43 antibody for 60 min at room temperature. The wells were then incubated with neutravidin-peroxidase (1/600) (Gibco BRL) for 30 min. The reaction was detected using a chemiluminescent substrate (SuperSignal West Pico, Pierce Chemical Co.). The chemiluminescence measurement was performed using a microplate luminometer (Series 7700, VER-4.03, Cambridge Technology, Inc). In inhibition assays, the reaction of coated biotinylated anti-paracoccin or anti-gp43 antibody with laminin-coated plates was carried out in the presence of 10 mM d-mannose or 10 mM *N-*acetyl-glucosamine.

### Measurement of production of TNF-α and nitric oxide by macrophages

We compared the ability of the fully glycosylated (PbAg) and under-glycosylated (PbAgTM) crude extracts to induce TNF-α and nitric oxide (NO) production in murine macrophages. C57BL/6 mice were stimulated with thioglycollate and cells were harvested from the peritoneal cavity. Adherent cells (macrophages) were stimulated for 48 h with PbAg, PbAgTM, or LPS+IFN-γ, in RPMI 1640 medium supplemented with 10% heat-inactivated fetal bovine serum and (100 mg/mL) streptomycin/gentamycin (Gibco). The culture was maintained at 37°C in a humidified atmosphere containing 5% CO_2_. The levels of TNF-α in the macrophage culture supernatants were measured by ELISA OptEIA™ Set for mouse (Pharmingen), as previously described [20], [21]. Cytokine TNF-alpha concentrations were determined from a standard curve for serial 2-fold dilutions of recombinant murine TNF-α. The amount of NO in the macrophage culture supernatants was quantified by analyzing the accumulation of nitrite in the supernatant monolayer by using the standard Griess reaction, as previously described [20], [21].

### Statistical analysis

Data are either the means of or representative results from at least 3 independent experiments, each performed in triplicate. Statistical analysis and comparisons were performed using GraphPad Prism Software version 5.00. The one-way ANOVA and Turkey's multiple comparison post test were applied. A p of <0.05 was considered statistically significant.
